# Senescent Mesenchymal Stem Cells in Myelodysplastic Syndrome: Functional Alterations, Molecular Mechanisms, and Therapeutic Strategies

**DOI:** 10.3389/fcell.2020.617466

**Published:** 2021-02-11

**Authors:** Xiaofang Chen, Ningyu Li, Jianyu Weng, Xin Du

**Affiliations:** ^1^Department of Hematology, Guangdong Provincial People's Hospital, Guangdong Academy of Medical Sciences, Guangzhou, China; ^2^School of Medicine, South China University of Technology, Guangzhou, China

**Keywords:** mesenchymal stem cells, senescence, myelodysplastic syndrome, bone marrow microenvironment, treatment

## Abstract

Myelodysplastic syndrome (MDS) is a group of clonal hematopoietic disorders related to hematopoietic stem and progenitor cell dysfunction. However, therapies that are currently used to target hematopoietic stem cells are not effective. These therapies are able to slow the evolution toward acute myeloid leukemia but cannot eradicate the disease. Mesenchymal stem cells (MSCs) have been identified as one of the main cellular components of the bone marrow microenvironment, which plays an indispensable role in normal hematopoiesis. When functional and regenerative capacities of aging MSCs are diminished, some enter replicative senescence, which promotes inflammation and disease progression. Recent studies that investigated the contribution of bone marrow microenvironment and MSCs to the initiation and progression of the disease have offered new insights into the MDS. This review presents the latest updates on the role of MSCs in the MDS and discusses potential targets for the treatment of MDS.

## Introduction

Myelodysplastic syndromes (MDS) lead to a clonal disease of the hematopoietic system, characterized by ineffective hematopoiesis and a high risk of transforming into acute myeloid leukemia (AML).

In most studies, MDS is considered a hematopoietic cell disorder in which disease initiation and progression are exclusively driven by hematopoietic cell-intrinsic genetic events. During the past 10 years, a large number of studies have been conducted on the genetic and molecular aspects of cloned cells in MDS, and more than 40 gene mutations associated with the prognosis of patients, as well as treatment targets, have been revealed. However, these mutations are not specific and have been found in normal people as well as patients with idiopathic cytopenia of undetermined significance (ICUS), where a certain proportion of the latter population eventually develops MDS (Glenthøj et al., 2016). Therefore, the mechanisms underlying MDS initiation and progression cannot be fully attributed to genetic and molecular changes alone. Several earlier observations have challenged this reductionist view, and a large number of studies have shown that MDS is associated with an abnormal bone marrow (BM) microenvironment.

The post-birth hematopoietic microenvironment is mainly located in the BM and comprises interstitial cells, helper cells, and sympathetic nerve cells. Bone abnormalities such as “adynamic” bone, characterized by reduced osteoblast numbers, decreased mineral apposition rates, and osteoporosis, have been noted in MDS patients in comparison with age-matched controls (Mellibovsky et al., 1996; Weidner et al., 2017). Mesenchymal stem cells (MSCs), which are a source of stromal cells in the hematopoietic environment, have subsequently been identified as key components of this disrupted architecture. *Ex vivo*-expanded MSCs display altered differentiation characteristics, transcriptional abnormalities, and a reduced ability to support hematopoietic stem/progenitor cells (HSPCs) in MDS, suggesting that MSCs may play a potential role in BM failure seen in MDS (Raaijmakers, 2012; Li and Calvi, 2017). MSCs play a crucial role in the BM microenvironment (Kfoury and Scadden, 2015; Pleyer et al., 2016). These cells display a potential for self-renewal and multidirectional differentiation and may differentiate into a variety of mesenchymal cells, such as osteoblasts, adipocytes, and chondrocytes. Precise regulation of hematopoietic stem cells (HSCs) maintains hematopoiesis for life. In addition, MSCs also display immunoregulatory functions, by maintaining the stability of the BM immune microenvironment and reducing the damage caused to HSCs by stress stimuli. Animal studies have shown that genetic abnormalities in MSCs are sufficient to induce MDS formation. Dysfunctional MSCs also play an important role in the progression of MDS and its transformation to AML.

At present, the understanding of genetics and gene expression characteristics associated with MDS-derived MSCs remains limited, and experimental results pertaining to the morphology, proliferation, differentiation, and hematopoiesis show inconsistencies. However, the association between functional changes in aging MDS-MSCs and disease progression, as well as the relevance of such changes to prognostic evaluation and treatment, is increasingly attracting the attention of researchers. Thus, a better understanding of the role played by MSCs in the pathogenesis of MDS may help strengthen knowledge regarding the complexity of MDS pathogenesis and help determine new treatment options.

This review describes current target-HSC treatments and elaborates on the role of the BM microenvironment in MDS. The importance of MSC senescence and phenotypic characteristics, as potential targets for MDS treatment, is discussed. This review may help improve existing knowledge regarding the initiation and progression of MDS and enable new targets for the treatment of MDS to be determined.

## Current Status of MDS Treatment

Treatment of MDS, which is based on the WHO Prognostic Scoring System (WPSS) and the International Prognostic Scoring System (IPSS and IPSS-R) for risk stratification, involves individualized treatment measures (Ferrer et al., 2013). For the MDS treatment algorithm, all patients should receive appropriate supportive treatment. Since then, the MDS expert group proposed to initially divide patients with clinically significant cytopenias into two main risk groups: (A) lower-risk patients [including IPSS low, intermediate-1; IPSS-R very low, low, and intermediate (≤3.5); and WHO-based Prognostic Scoring System for risk stratification (WPSS) very low, low, and intermediate] and (B) higher-risk patients [including IPSS intermediate-2 and high; IPSS-R intermediate (>3.5), high, and very high; and WPSS high and very high]. In addition, intermediate-risk patients with disease that does not respond to therapy for lower-risk disease would be eligible to receive therapy for higher-risk MDS. In addition, intermediate-risk patients who do not respond to treatment of lower-risk diseases are eligible for treatment of higher-risk MDS.

**Figure 1:**
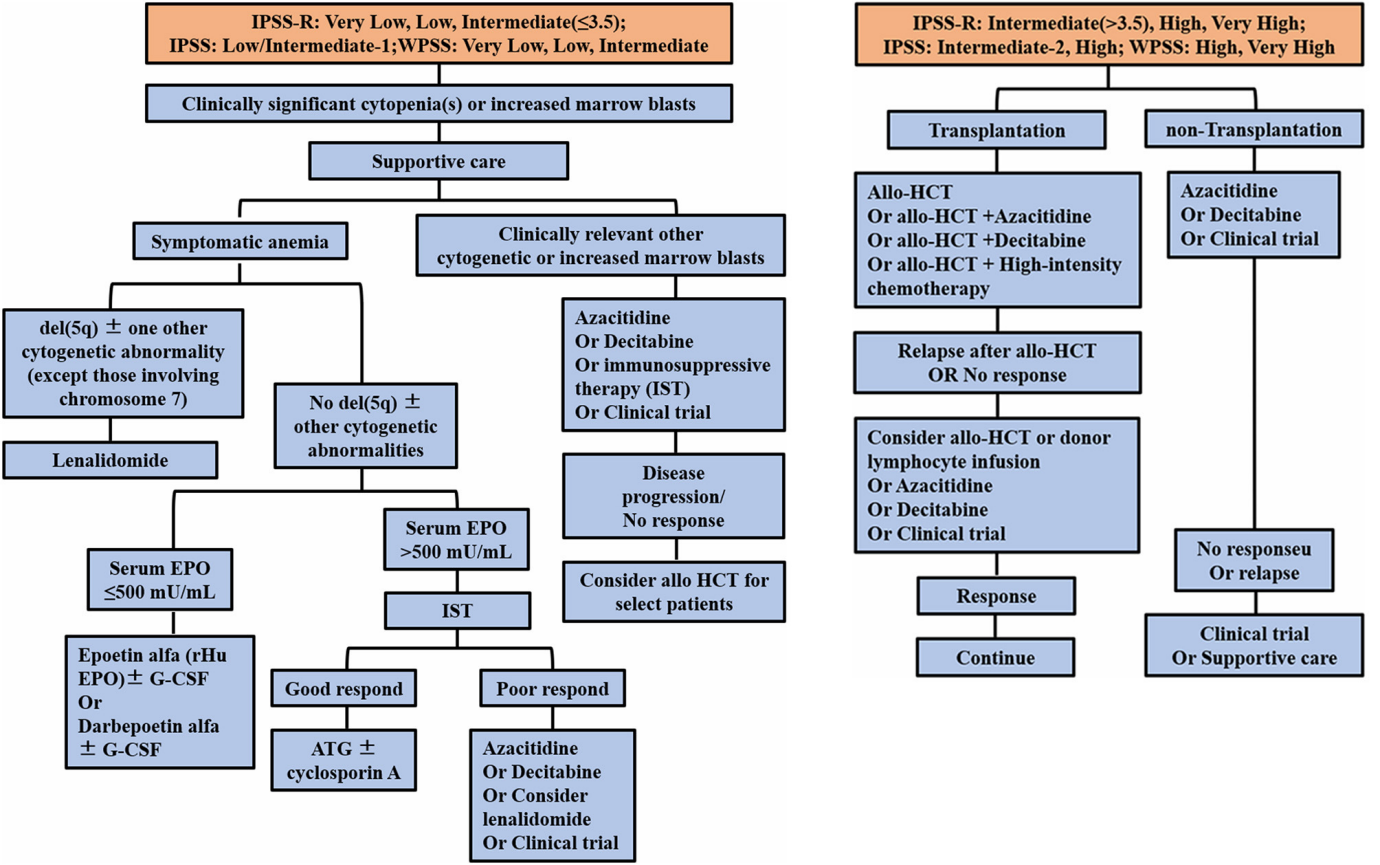
Current treatment of MDS, based on the IPSS and revised IPSS and WHO-based Prognostic Scoring System for risk stratification (WPSS). EPO, erythropoietin; G-CSF, granulocyte colony-stimulating factor; IST, immunosuppressive therapy; HCT, hematopoietic cell transplantation.

Patients with low-risk MDS are provided with supportive treatment using cytokines and immunomodulatory drugs, such as lenalidomide. Patients with del(5q) (5q^−^) chromosomal abnormalities with or without one other cytogenetic abnormality, except those involving chromosome 7 and symptomatic anemia should be treated with lenalidomide. Studies have shown the relative safety of lenalidomide in these patients and the improved quality-of-life (QOL) outcomes in randomized clinical trials (Oliva et al., 2013; Revicki et al., 2013). Patients without the 5q^−^ abnormality, with or without one other cytogenetic abnormality and accompanied by symptomatic anemia, are categorized by serum erythropoietin (EPO) levels. These patients should receive erythropoietin, G-CSF, or iron repletion treatment (Hellström-Lindberg, 1995; Negrin et al., 1996; Hellström-Lindberg et al., 1997; Casadevall et al., 2004; Spiriti et al., 2005) (Figure 1). Patients without symptomatic anemia, with increased BM blasts or any other clinically relevant cytopenias, should be considered for treatment with decitabine, azacytidine, immunosuppressive therapy, or a clinical trial (Jabbour et al., 2017).

Patients with high-risk MDS are administered demethylating drugs, such as decitabine and azacytidine, or allogeneic HSC transplantation, which is considered the only possible cure for MDS (Figure 1). However, due to limitations associated with the application of transplantation technology to MDS patients, most patients are found to be unsuitable for this treatment program (Alessandrino et al., 2008; Hicks et al., 2013; Oliva et al., 2013; Revicki et al., 2013). In therapeutic trials, the panel, using the standardized International Working Group (IWG) response criteria for evaluating studies, found it important to stipulate that all MDS patients should be given relevant supportive care (Cheson et al., 2000, 2006; Hicks et al., 2013; Pfeilstöcker et al., 2016). The latest developments pertaining to MDS treatment during the past 10 years indicate that lenalidomide and decitabine may improve the hematology of low-risk and high-risk patients, respectively, and that azacytidine is the only drug that may prolong the overall survival of high-risk MDS patients (Silverman et al., 2011). However, lenalidomide does not delay the progression of patients to high-risk MDS and subsequent conversion to AML. Failure of MDS treatments to achieve a breakthrough may be due to the fact that genetic and molecular abnormalities in the cloned cells associated with MDS do not clearly relate to events leading to the onset of MDS or to disease progression.

Therefore, new drugs developed to counter different molecular mechanisms related to MDS-cloned cells have been largely ineffective. These results indicated that other mechanisms may be involved in the initiation and progression of MDS. Mouse MDS models, such as Dicer1-knockout and NHD13 mice, exhibited MSC dysfunction and MDS-like morbidity hematopoiesis, which play an important role in the progression of MDS disease (Lin et al., 2005; Balderman et al., 2016). These results indicated that the dysfunctional MSCs derived from MDS are related to the pathogenesis of MDS.

## The Role of BM Microenvironment in MDS

It has long been considered that hematopoietic cell disorders, which are solely driven by genetic events, are capable of inducing the initiation and progression of MDS. However, several previous studies have suggested that the BM microenvironment also contributes to MDS pathogenesis (Verstovsek et al., 2002; Iwata et al., 2007; Ferrer et al., 2013; Geyh et al., 2013). The BM microenvironment plays an essential role in the maintenance and development of HSPCs. It regulates quiescence, self-renewal, proliferation, and differentiation of stem cells (Adams and Scadden, 2006; Li and Li, 2006; Moore and Lemischka, 2006). The cells in the BM microenvironment are dynamic in nature and may shape the environment to favor an abnormal population of niche residents *via* certain mechanisms. Colmone et al. reported that the interaction between normal HSPCs and the BM was influenced by a leukemia cell line (Colmone et al., 2008). More definitively, genetic changes in hematopoietic cells cause secondary changes in the cells constituting the BM microenvironment, which supports abnormal hematopoietic populations (Schepers et al., 2013). Bone abnormalities in MDS patients such as “adynamic” bone, displayed decreased osteoblast numbers and osteoporosis, compared to age-matched controls (Mellibovsky et al., 1996; Weidner et al., 2017).

Prior to hematopoietic cell transplantation, chemotherapy and radiation therapy were used to exert direct effects on HSCs and the microenvironment and markedly changed the normal supportive BM environment (Barcellos-Hoff et al., 2005; Wright, 2005). Mice that were irradiated following transplantation of growth factor-dependent syngeneic mouse BM cells developed leukemia at a faster rate and in higher numbers compared with non-irradiated recipients (Dührsen and Metcalf, 1990). Thus, in allogeneic patient BM environments, normal transplant-donor cells are transformed into hematopoietic neoplastic cells, demonstrating that the BM environment may contribute to the initiation of MDS (Flynn and Kaufman, 2007). Although infrequent, it appears that interaction between the BM microenvironment and genetically aberrant HSPCs may induce MDS pathogenesis. Thus, during the last decade, the BM microenvironment has been shown to exhibit a potentially permissive or causative role, challenging the belief that hematopoietic cell disorder is solely an element of the initiation and progression of MDS.

## MSC Senescence and Phenotypic Characteristics in MDS

**Figure 2:**
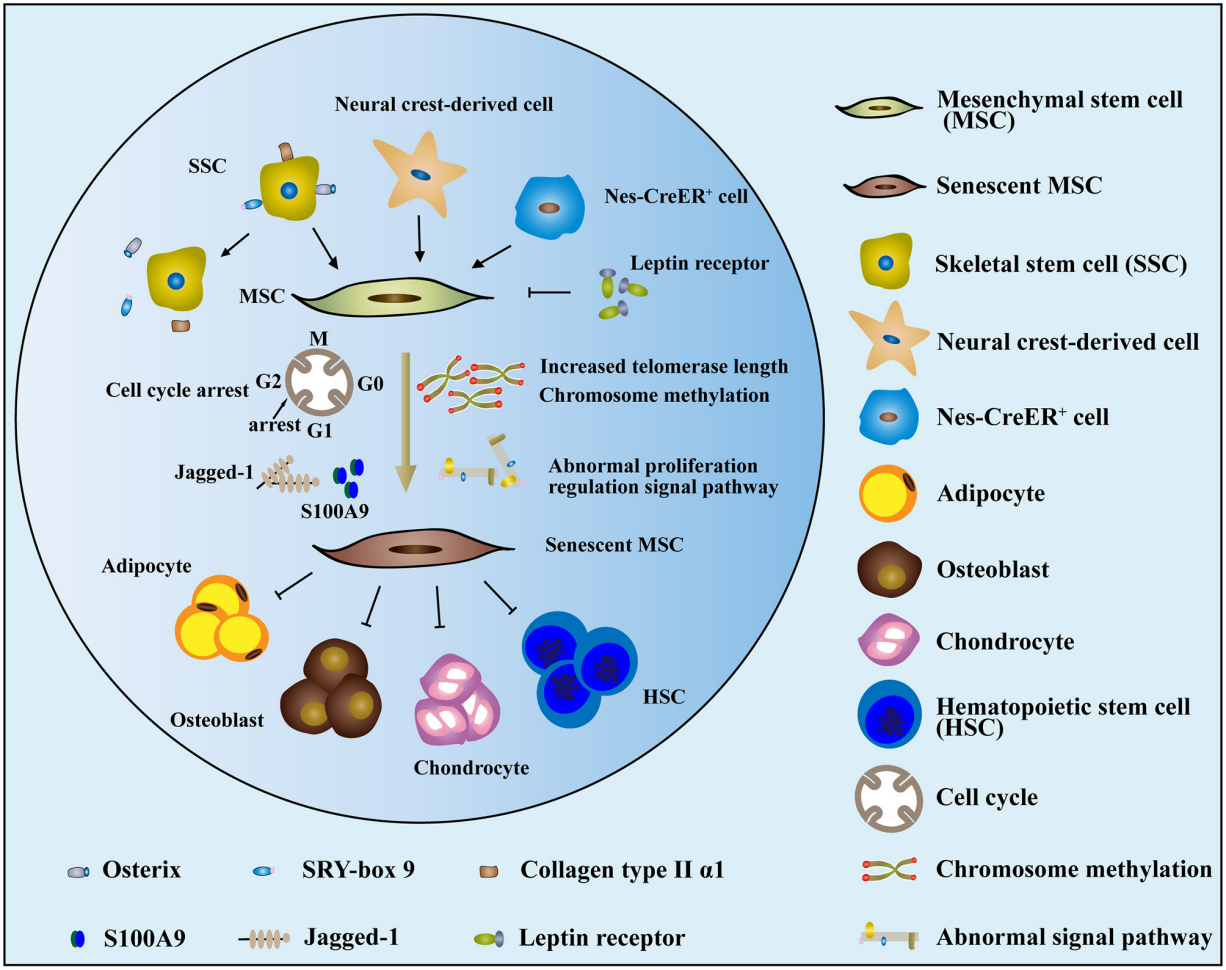
The mechanism and dysfunction of MSC senescence in MDS. (1) Cells that contributed to MSCs, such as SSCs, neural crest-derived cells, and nes-CreER+ stromal cells, were reduced or depleted in MDS. In contrast, leptin receptors, which are detrimental to the function of MSCs, increased over time. (2) The senescence of MDS-MSCs may be related to increases in cell telomerase length, chromosome methylation status, abnormal proliferation regulation signal pathway, cell cycle arrest, and other factors. (3) The senescent MDS-MSCs exhibited reduced differentiation potential and stem cell support capacity.

MSCs, which display pluripotent and undifferentiated capacities, are key components of the BM microenvironment (Sacchetti et al., 2007). In recent years, evidence has increasingly demonstrated that MSCs in MDS are intrinsically pathological and that senescence is increased by a continuous decline in proliferation (Ferrer et al., 2013; Geyh et al., 2013). Starting from birth and proceeding into adulthood, the composition of BM stromal cells changes over time. MSCs and HSCs/HSPCs that colonize adult BM during development undergo changes in composition over time. During later stages of fetal development, skeletal stem cells (SSCs), which are characterized by the expression of *Osx* (encoding osterix), *Sox9* (encoding SRY-box 9), and *Col2a1* (encoding collagen type II α1 chain), produce bone and BM stromal cells, which persist postnatally (Maes et al., 2010; Mizoguchi et al., 2014; Ono et al., 2014b). However, as depicted in Figure 2, SSCs are not labeled by these markers, while stromal cells are depleted during adulthood (Maes et al., 2010; Park et al., 2012; Mizoguchi et al., 2014; Ono et al., 2014b). Neural crest-derived cells make a short-term contribution to BM stromal cells and the activity of colony-forming unit fibroblasts (CFU-Fs) during the early postnatal period, but these cells decrease in adulthood too and are replaced by nonneural crest-derived stromal cells (Takashima et al., 2007; Komada et al., 2012; Isern et al., 2014). Consistent with this, Nes-CreER^+^ (*Nes* encodes nestin) stromal cells promote bone formation in early postnatal BM but exert little or no effect on osteogenesis or CFU-F activity in adult BM (Figure 2) (Ono et al., 2014a; Zhou et al., 2014). By contrast, leptin receptor-expressing cells appear in the BM postnatally and exert an effect on the stromal cell population or osteogenesis. As numbers increase, these become the main source of adipocytes and osteoblasts in adults over time (Figure 2) (Méndez-Ferrer et al., 2010; Zhou et al., 2014).

Cellular senescence, a complex process that is usually accompanied by functional changes, is a special state of cell cycle arrest in proliferating cells under the stimulation of stress factors, wherein cells undergo a series of changes in morphology, proliferation, differentiation, secretion, and other functional abnormalities (Pleyer et al., 2016). Changes in morphology intuitively reflect the phenomenon of senescence in MDS-MSCs. MSCs in the donor group are usually slender fibers or fusiform, whereas the volume of MDS-MSC is obviously increased, showing a flat irregular polygon. A cytoskeletal morphology study showed that the morphological changes in MDS-MSCs were related to increased, as well as disorderly F-actin distribution. The expression level of β-galactosidase is significantly increased in senescent cells. Staining experiments indicated that β-galactosidase in MDS-MSCs was significantly increased, directly illustrating the aging of MSCs in MDS (Ferrer et al., 2013; Geyh et al., 2013; Fei et al., 2014; Zhao et al., 2015). Senescence of MDS-MSCs also manifests as a decline in proliferation ability. Parameters such as CFU-F, cumulative number of passages, and doubling time were significantly worse in the MDS-MSC group compared to those of control MSCs. The proliferation ability of MDS-MSCs was significantly reduced. Such a decrease in MSC proliferation capacity may be related to increases in cell telomerase length, chromosome methylation status, abnormal proliferation regulation signal pathway, cell cycle arrest, and other factors (Pavlaki et al., 2014; Falconi et al., 2016).

Decreased differentiation in aging MDS-MSCs both *in vivo* and *in vitro* was associated with the senescence and aging of MSCs (Stenderup et al., 2003; Bonab et al., 2006; Zhou et al., 2008). Alizarin Red chemical staining showed that the osteogenic differentiation potential of MDS-MSCs decreased significantly during the *in vitro* induction of differentiating MSCs into osteoblasts (Ferrer et al., 2013; Geyh et al., 2013; Fei et al., 2014; Zhao et al., 2015). The expression levels of genes involved in osteogenic differentiation accurately indicated the differences between the osteogenic differentiation potentials of cells. The transcription factors, RUNX2 and Osterix, involved in regulating early osteogenic differentiation and genes encoding osteocalcin, serve as mature osteogenic markers in MDS-MSCs. The expression levels of these were significantly lower than those of the control group, indicating that the osteogenic differentiation potential of MSCs in aging MDS had decreased (Ferrer et al., 2013; Geyh et al., 2013; Fei et al., 2014; Zhao et al., 2015).

Senescent cells still show metabolic activity, but many cytokines associated with senescence in MDS-MSCs, such as transforming growth factor β1 (TGFβ1), HGF, Jagged1, angiopoietin-1, osteopontin, CXCL-12, IL-6, TGF-β, SCF, and VEGF, are expressed abnormally. Jagged1 is a ligand involved in the Notch signaling pathway, the activation of which plays an important regulatory role in the differentiation of HSCs and the formation of myeloid tumors (Geyh et al., 2013; Pavlaki et al., 2014). The expression of *jagged-1* in MDS-MSCs was significantly increased, and 38% of MDS tumor cells showed excessive activation of the Notch signaling pathway (Geyh et al., 2013; Pavlaki et al., 2014). S100A9 promotes cellular senescence of BM stromal cells via TLR4, NLRP3 inflammasome formation, and IL-1β secretion (Shi et al., 2019). Some reports have indicated that MSCs exhibited insufficient hematopoietic support capability in MDS compared with donor cells (Zhao et al., 2012; Ferrer et al., 2013). Finally, MSCs from MDS were more prone to cellular senescence than donor MSCs, where senescent MDS-MSCs exhibited reduced differentiation potential and stem cell support capacity.

## Changes in the Function of Aging MDS-MSC to Support Hematopoiesis

As described above, *in vivo* mouse experiments have demonstrated that abnormal MSCs may affect the initiation and progression of MDS. Knocking out *Dicer1*, which encodes endonuclease III of miRNA in mouse MSCs, caused MDS-like morbid hematopoiesis in the BM, transforming a portion of the mouse population into AML (Kfoury and Scadden, 2015). Several mouse models have been developed to mimic human MDS, of which the mouse NUP98-HOXD13 (NHD13) transgenic model, wherein MSCs and osteoblast dysfunction play an important role in MDS progression, may be the most accurate (Lin et al., 2005; Balderman et al., 2016). Following transplantation of NHD13 mouse hematopoietic cells into both NHD13 and WT mice, the rates of AML transformation and mortality in NHD13 mice were found to be significantly higher than those in WT mice. By contrast, hematopoiesis of NHD13 mice transplanted with BM from WT mice showed a myeloid bias, while the hematopoietic function of WT mice was impaired due to dysfunctional MSCs (Balderman et al., 2016).

Abnormal functioning of proliferation, differentiation, and secretion of aging MDS-MSCs leads to changes in the regulation of hematopoietic function. In an *in vitro* co-culture experiment using MDS-MSCs and donor HSCs, the proportion of hematopoietic cells in the G1 phase decreased while the proportion in the G0 phase increased, in a manner which was significantly different from that in normal MSCs (*P* = 0.0076) (Geyh et al., 2013). MDS-MSCs showed a significantly altered cell cycle status and displayed a shift toward increased apoptosis compared to control MSCs. These changes may contribute to the pathogenesis of MDS (Abbas et al., 2019).

High levels of hyaluronan (HA) were detected in the BM sera of higher-risk MDS patients in comparison with those of donor controls. High levels of HA in BM serum, which enhances osteogenic differentiation of MSCs, were associated with adverse clinical outcomes and significantly shorter median survival in MDS (Fei et al., 2018).

Defective proliferation was observed in pediatric MDS-derived MSCs. Pediatric MDS-derived MSCs were more prone to cellular senescence than healthy controls and showed a decrease in the S phase (Liu et al., 2015). Iron overload (IO) reportedly promotes mitochondrial fragmentation and enhances autophagy in MSCs of MDS patients by activating the AMPK/MFF/Drp1 pathway (Zheng et al., 2018). These results indicated that dysfunctional MSCs derived from MDS were associated with MDS-associated pathogenesis.

## MSCs as A Potential Target for MDS Treatment

The functions of MDS-MSCs not only play an important role in the initiation and progression of the disease but also provide a protective microenvironment for tumor cells, which is a poor prognostic factor of MDS. Improving MDS-MSC function as an option for MDS treatment has gradually advanced from its theoretical basis to the research level. Co-cultivation experiments using HSCs *in vitro* have shown that MDS-MSCs inhibited erythroid hematopoiesis and promoted myeloid cell production, significantly restoring the contention that MDS-MSCs support erythroid hematopoiesis following lenalidomide treatment (Geyh et al., 2013). Demethylation drugs may simultaneously affect the functions of cloned cells and MSCs in MDS. Following transplantation of NHD13 mouse hematopoietic cells into normal mouse BM, the rates of transformation to acute leukemia and death were significantly reduced, while hemoglobin and white blood cell indicators in peripheral blood were enhanced (Balderman et al., 2016). These data indicated that improving MSC function may delay disease progression and enhance cytopenia of MDS.

However, the genetic and molecular changes occurring in MDS cloned cells do not fully clarify the initiation of MDS and disease progression. This may be an important factor affecting the treatment of hematopoietic cells, where most patients will eventually show a declining treatment response. Thus, treatment of MDS cloned cells alone may not produce a cure. However, intervention involving clonal cells and the BM microenvironment may lead to a new treatment strategy for MDS.

MDS-MSCs showed increased production of pro-inflammatory cytokines. Treatment with 5-azacytidine significantly decreased IL-6 levels in MDS-MSCs *in vitro*, compared to the IL-6 levels in MSCs from the donor. As MSCs produce much more inflammatory cytokines involved in MDS pathogenesis, these may represent a potential therapeutic target. Moreover, 5-azacytidine may exert a stromal effect, thereby modulating the immune response in MDS (Boada et al., 2020).

Following the administration of α-lipoic acid (ALA), the levels of reactive oxygen species (ROS) in MSCs were gradually decreased, intracellular iron content was reduced, and the potential and integrity of the mitochondrial membrane were restored. ALA treatment resulted in a significant decrease in autophagy, whose factor may be used against MDS (Camiolo et al., 2019). BM-MSCs showed improved proliferation activity in MDS patients. CDKN2A shows potential as a therapeutic target in regulating the BM microenvironment because early senescence is reversible *via* de-induction of CDKN2A (Choi et al., 2020).

Menatetrenone treatment of BM-MSCs enhanced CD34+ cell generation in cocultures by accelerating the cell cycle. MDS-derived cells underwent apoptosis when co-cultured with BM-MSCs, an effect that was enhanced by menatetrenone. These findings indicated that pharmacological treatment with menatetrenone bestows a unique hematopoiesis-supportive capability on BM-MSCs, which may contribute to clinical improvement of cytopenia in MDS (Fujishiro et al., 2020).

Upon exposure to TGFβ1, healthy MSCs developed functional deficits and adopted a phenotype similar to what was observed in patient-derived stromal cells. These suppressive effects of TGFβ1 on stromal cell functionality were abrogated by SD-208, an established inhibitor of TGFβ receptor signaling. Blockade of TGFβ signaling by SD-208 also restored the osteogenic differentiation capacity of patient-derived stromal cells (Geyh et al., 2018).

All such changes in MDS-MSCs may provide potential targets for the treatment of MDS.

## Conclusion

In brief, an increasing number of studies have shown that MDS is a group of heterogeneous diseases caused by abnormalities in both hematopoietic cells and the microenvironment. Tumor hematopoietic cells alter the function of the hematopoietic cell microenvironment *via* direct contact and secretion of cytokines, causing it to be conducive to the growth of tumor cells. A change in the number and function of MSCs leads to a decline in the supporting function of normal hematopoietic cells, by providing a protective microenvironment for tumor cells, which is conducive to the proliferation of tumor cells. This confers proliferation advantages to tumor cells and reduces tumor cell sensitivity to chemotherapeutic agents and drugs. Gender may be an important reason for the widespread resistance to the disease. If large-sample clinical trials indicate that changes in the number and function of MSCs may be used as potential biomarkers for predicting patient response to treatment or recurrence following therapy, then the changes in MSCs at the time of diagnosis and during treatment will contribute to the formation of personalized therapies.

## Author Contributions

XC and NL searched the literature and drafted part of the manuscript. XD designed the entire study and revised the manuscript. JW revised the manuscript. All authors contributed to the article and approved the submitted version.

## Conflict of Interest

The authors declare that the research was conducted in the absence of any commercial or financial relationships that could be construed as a potential conflict of interest.
